# Clinical profile and outcomes of tetanus patients in a tertiary hospital in the Philippines: a ten-year retrospective study

**DOI:** 10.1186/s12879-024-09037-1

**Published:** 2024-01-29

**Authors:** Pamela Danielle T. Lanuza, Jao Jarro B. Garcia, Christian Wilson R. Turalde, Mario Jr. B. Prado

**Affiliations:** 1 Division of Adult Neurology, Department of Neurosciences, College of Medicine and Philippine General Hospital, University of the Philippines Manila, Manila, Philippines; 2 Department of Physiology, College of Medicine, University of the Philippines Manila, Manila, Philippines; 3 National Teacher Training Center for the Health Professions, University of the Philippines Manila, Manila, Philippines; 4 Department of Epidemiology and Biostatistics, College of Public Health, University of the Philippines Manila, Manila, Philippines

**Keywords:** Tetanus, Philippines, Risk factors, Outcomes

## Abstract

**Background:**

Tetanus is a life-threatening but preventable neurologic disorder characterized by trismus and muscle spasms. Despite its decreasing global incidence, it remains to be endemic in resource-limited settings such as the Philippines. This study aimed to determine the incidence, demographic characteristics, risk factors, clinical presentation, management, complications, and outcomes of non-neonatal tetanus cases in a tertiary hospital in the Philippines. It also aimed to compare the clinical profile and outcomes between the adult and pediatric subgroups.

**Methods:**

This study used a retrospective cross-sectional design including all adult and pediatric non-neonatal tetanus patients admitted at the University of the Philippines - Philippine General Hospital from January 2012 to June 2023. Data was extracted from department censuses and inpatient charts.

**Results:**

One hundred thirty-eight cases were included. The incidence rate was 0.03%, while mortality rate was 29%. Majority of patients were males presenting with trismus and spasms after sustaining a puncture wound. Chronic hypertension was associated with an increased hazard of death by 4.5 times (*p* = 0.004), while treatment with magnesium sulfate was associated with a decreased hazard of death by 35 times (*p* = 0.005). The mode of infection and the medications administered differed between the adult and pediatric subgroups.

**Conclusions:**

Although the total number of cases has decreased over the past decade, tetanus remains to have a high incidence and mortality rate in the Philippines. Increasing vaccination coverage, improving public awareness, and educating health professionals can help reduce morbidity and mortality from this disease.

## Introduction

Tetanus is a potentially life-threatening but preventable neurologic disorder characterized by generalized rigidity and painful muscle spasms caused by tetanospasmin, an exotoxin produced by the anaerobic bacterium *Clostridium tetani*. High-risk populations include unvaccinated individuals, intravenous drug users, surgical including burn and trauma patients, and the immunosuppressed. The incubation period of tetanus averages eight days, but may range from one to 21 days. Clinically, patients typically present with trismus (lockjaw) and risus sardonicus (rigid smile), progressing to neck stiffness, dysphagia, board-like abdominal rigidity, and opisthotonos (backward arching and extensor posturing). The diagnosis of tetanus is primarily clinical. Management includes neutralization of unbound toxins with immunoglobulin, wound debridement, antibiotic therapy, and control of muscle spasms with benzodiazepines and neuromuscular blockers [1–3].

The World Health Organization (WHO) reports a significant decline in the global incidence of tetanus from 36 per 1 million total population in 1982 to 0.9 per 1 million total population in 2022, owing to increasing vaccination coverage [4]. Majority of tetanus cases and deaths occur in children under 5 years of age, although this proportion has decreased from 78 to 53% over the past 30 years [5]. While less than 1 per million total population remain in developed regions, tetanus remains to be endemic in resource-limited countries including the Philippines. The Philippines reports an incidence rate of 6.9 tetanus cases per 1 million total population as of 2022; it remains to be a public health concern which poses a significant risk of morbidity and mortality [6].

To our knowledge, there are no recently published local data on the clinical profile and outcomes of both adult and pediatric non-neonatal tetanus patients. This study aims to (1) determine the incidence of non-neonatal tetanus in a tertiary hospital in the Philippines over the past 10 years, (2) determine their demographic characteristics, (3) identify risk factors predisposing them to develop tetanus, (4) describe their clinical presentation in terms of source of infection, symptoms, and disease severity, (5) describe their management in the local setting, (6) describe their clinical course including complications encountered, (7) determine their outcomes and identify the leading causes of mortality, and (8) differentiate the clinical profiles and outcomes of adult and pediatric patients.

## Methods

### Study design and setting

This study utilized a retrospective cross-sectional study design involving all non-neonatal tetanus patients admitted at the University of the Philippines - Philippine General Hospital (UP-PGH) from January 2012 to June 2023. The UP-PGH is the largest modern government tertiary hospital and the only national referral center for tertiary care in the country, serving over 600,000 patients annually [7].

### Study population

#### Case identification and sampling

Cases were identified using a review of the censuses of the UP-PGH Departments of Neurosciences, Pediatrics, and Surgery from January 1, 2012 to June 30, 2023. All cases which fulfilled the inclusion criteria listed below were included in the study.

#### Inclusion and exclusion criteria

Filipino patients more than 28 days old, who were clinically diagnosed by a physician as a case of tetanus and fulfilled the WHO case definition of suspected non-neonatal tetanus with acute onset of trismus, risus sardonicus, or generalized muscle spasms, were included. Patients whose clinical presentations were better explained by an alternative diagnosis were excluded.

### Data collection

Access to physical and electronic inpatient records was requested from the UP-PGH Medical Records Division. Chart review was performed by two independent reviewers (PTL, JSP) to ensure completeness of data collection. Data was encoded into a pre-made detailed abstraction form containing the variables of interest using unique patient codes.

Demographic characteristics such as age, sex, and place of residence, as well as comorbidities (including malignancy, cardiovascular, endocrine, neurologic, pulmonary, renal, and hepatic diseases, and immunocompromised states) were recorded. Data on educational attainment, occupation, socioeconomic status, and vaccination status were supposed to be included, but were generally unavailable. In terms of clinical presentation, presumed source of infection, type of injury sustained, site of injury, interval from injury until onset of symptoms, presenting symptoms, tetanus type, and severity staging were recorded. In terms of management, medications (such as antibiotics, benzodiazepines, neuromuscular blockers, and magnesium sulfate) and subspecialties involved in patient care were noted. Data on the clinical course was recorded in terms of duration of hospital and intensive care unit (ICU) stay, interval from admission to tracheostomy, complications encountered (such as pneumonia, catheter-associated urinary tract infection, bacteremia, catheter-related bloodstream infection, acute kidney injury, embolic events, and pressure ulcers), and presence of autonomic dysfunction. Lastly, outcome of admission and cause of mortality, if applicable, were also included.

Data on the total number of hospital admissions from January 2012 to June 2023 was obtained from the UP-PGH Institutional Research, Planning, and Development Staff (IRPDS).

### Data analysis

All patients fulfilling the inclusion criteria were included. Continuous variables were presented using means or medians with their accompanying standard deviations or range respectively while categorical variables were presented as proportions. Differences between the pediatric and adult groups were compared using unpaired t-test or Wilcoxon-Rank Sum test for continuous variables, or Fisher exact test or chi-square test for proportions whichever was applicable. To determine the factors associated with survival, cox regression analysis was used using death as event variable, duration from time of admission to event as time variable and factors with p-value of less than 0.2 (pediatric vs. adult) as independent variables. A Kaplan Meier Curve using age (pediatric vs. adult) as strata with log rank test to determine difference was likewise created. All analysis was done using STATA 18 BE and p-value of 0.05 as cut off.

### Ethical considerations

This study adhered to the Philippine National Ethical Guidelines for Health and Health-Related Research (NEGHHR) 2017 and was carried out in accordance with the Declaration of Helsinki. The protocol was approved by the UP-PGH Department of Neurosciences Technical Review Board and the UP Manila Research Ethics Board (UPMREB 2023-0345-01). The principal investigators have no conflict of interest to declare in this study.

## Results

### Baseline characteristics

There were 138 non-neonatal tetanus cases (98 adult and 40 pediatric) identified upon census review from January 2012 to June 2023, all of which were included in this study. Eighty-two charts (55 physical and 27 electronic) were retrieved. The remaining 56 physical charts (37 adult and 19 pediatric) were either unavailable or disposed of as of writing, hence data collection was limited to information included in the censuses (i.e., age, sex, diagnosis, medications, outcome) for these cases. The clinical characteristics and outcomes of these tetanus patients are summarized in Table 1.

**Fig. 1:**
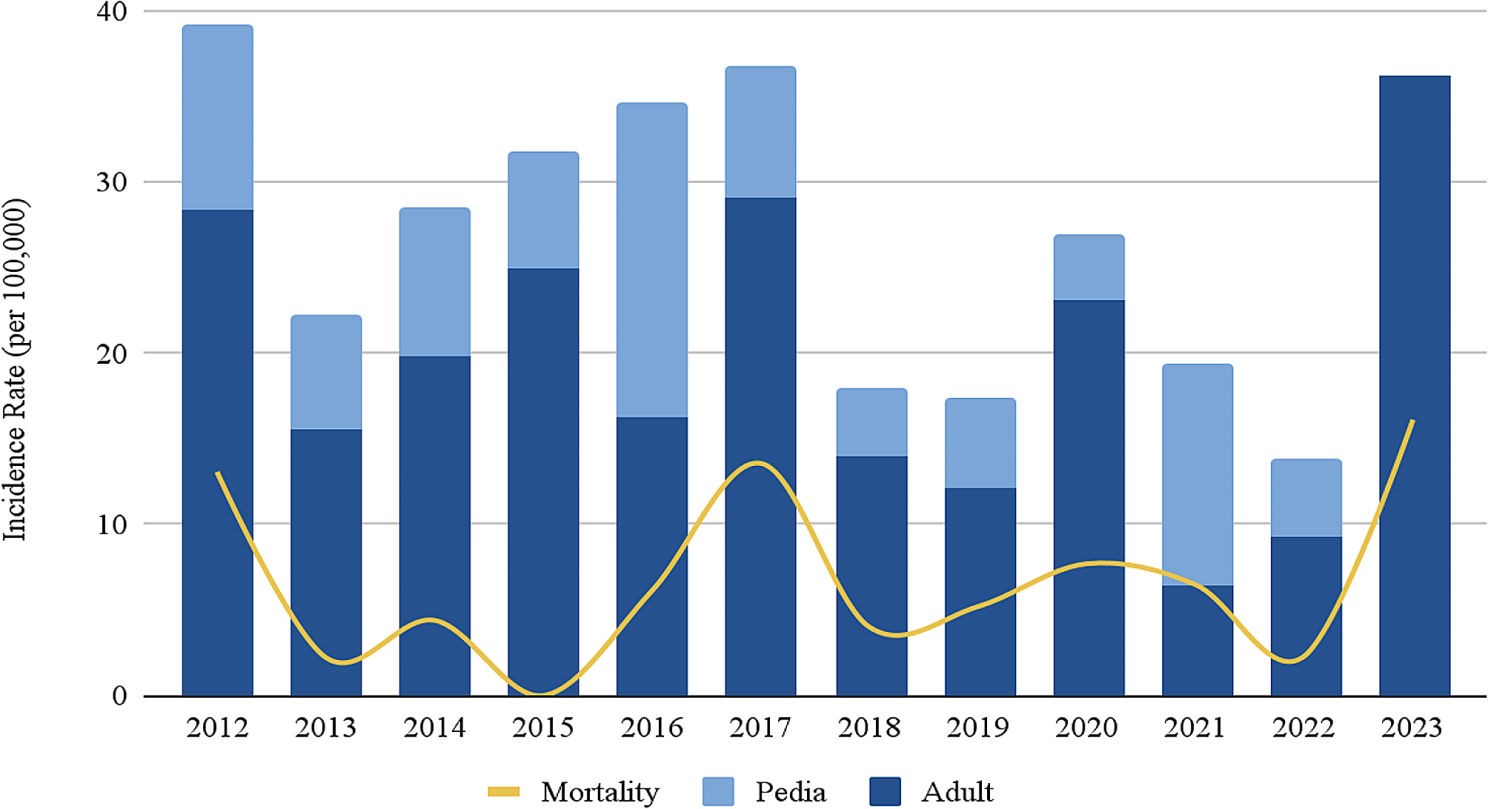
Trends in the incidence and mortality of adult and pediatric non-neonatal tetanus cases admitted in UP-PGH from January 2012 to June 2023

The cumulative incidence rate of non-neonatal tetanus in UP-PGH from January 2012 to June 2023 is 0.027% or 26.9 per 100,000 total inpatient admissions. Trends in the incidence of tetanus in UP-PGH compared with trends in the total number of tetanus cases in the Philippines are reported in Fig. 1.

The overall median age of tetanus patients was 37.5, 71% were adults (median: 47 years, range 20–75), while 29% were pediatric (median: 8 years, range: 2–18). Majority were males (74%, *n* = 102) residing in the CALABARZON region (47%, *n* = 41). Malignancy and hypertension were the most common comorbidities, which were present in 23% (*n* = 16) and 17% (*n* = 12) of adults respectively. Pulmonary disease, particularly COVID-19 pneumonia, was the only identified comorbidity in the pediatric subgroup (5%, *n* = 1). There was generally no information on tetanus vaccination status included in the patient records.

### Clinical presentation

Most patients diagnosed with tetanus had a history of trauma (49%, *n* = 45), while in a smaller proportion of cases, the presumed sources were non-traumatic infections, such as infected breast masses, non-healing foot ulcers, and ear infections (38%, *n* = 35). Puncture wounds were the most common type of injury sustained in adult patients (35%, *n* = 24), whereas head and neck infections were the most common in pediatric patients (32%, *n* = 7). The lower extremities were the most common site of injury and infection in adults (48%, *n* = 33), while the head region was most commonly affected in children (45%, *n* = 10). The median interval from the presumed antecedent event to the initial symptom was 7 days (range: 0-176 days), whereas the median interval from the initial symptom to the first spasm was 1 day (range: 0–28 days).

Trismus (or lockjaw) was the initial symptom noted in the majority of patients (65%, *n* = 55). Overall, the most common symptoms reported included trismus (99%, *n* = 83), spasms (86%, *n* = 72), dysphagia (58%, *n* = 49), rigidity (56%, *n* = 47), and neck stiffness (40%, *n* = 34). Spasms were most often noted in the trunk region (69%, *n* = 58). Most patients were diagnosed with generalized tetanus (86%, *n* = 113) in its most severe stage (58%, *n* = 76). The Cole, Ablett, and RITM severity staging systems were mentioned.

### Management

Anti-tetanus serum (ATS) was administered at a median dose of 10,000 units in adults and 3,000 units in children, either intramuscularly alone (80%, *n* = 66) or intramuscularly and infiltrated to the wound (13%, *n* = 11). Tetanus toxoid (TeAna) was given at a median dose of 0.5 ml. Metronidazole was the antibiotic of choice given to all patients (*n* = 91). Diazepam was given in 97% of adults (*n* = 69), while midazolam and rocuronium were the preferred agents in the pediatric subgroup, given in 95% (*n* = 19) and 85% (*n* = 17) of patients respectively. Magnesium sulfate, baclofen, and carbamazepine were other medications administered, with the latter two only given in adults. Among these medications, magnesium sulfate was found to reduce the hazard of death by 9 times (*p* = 0.005). When possible confounders and effect modifiers were controlled, magnesium sulfate was found to decrease the hazard of death by 35 times (*p* = 0.005) compared to patients who did not receive magnesium sulfate.

Adult Neurology (64%, *n* = 89), Pediatrics (28%, *n* = 39), and Surgery (3%, *n* = 4) served as the primary services in charge. Comanaging services included Otorhinolaryngology (90%, *n* = 76), Infectious Disease, (88%, *n* = 74), General Medicine (54%, *n* = 45), and Rehabilitation Medicine (48%, *n* = 40), with a median of 6 (range: 2–13) onboard services per patient.

### Clinical course and outcome

The median length of hospital stay was 19 days (range: 0-198), while the median duration of ICU stay was 10 days (range: 0–49). The median time from admission to tracheostomy was 0 or on the day of admission (range: 0-169). The median number of complications encountered during admission was 1 (range: 0–7). More than half of patients succumbed to nosocomial pneumonia (59%, *n* = 54). Other less common complications were bleeding from any cause (14%, *n* = 13), pressure ulcers (13%, *n* = 12), and bacteremia (13%, *n* = 12).

Majority of patients were discharged (67%, *n* = 78). Overall mortality rate was at 29% (*n* = 34); 27% (*n* = 24) and 36% (*n* = 10) for the adult and pediatric subgroups respectively.

### Differences between adult and pediatric cases

In terms of clinical presentation, puncture wounds (35% vs. 23%, *p* = 0.004) and involvement of the lower extremities as site of injury (48% vs. 23%, *p* = 0.023) were more common in adults than in children. There was no significant difference in terms of intervals from trauma to presenting symptom and from presenting symptom to first spasm between the two subgroups (*p* = 0.13 and *p* = 0.07 respectively).

Differences in terms of medications administered were also noted. Baclofen was only used in the adult population (*p* < 0.0001), while Midazolam (95% vs. 35%, *p* < 0.0001) and Rocuronium (85% vs. 11%, *p* < 0.0001) were used more in children. More services were involved in the care of adult patients (median of 6.5 vs. 4.9, *p* = 0.0031). There were no significant differences in terms of the complications encountered between the two subgroups.

**Fig. 2:**
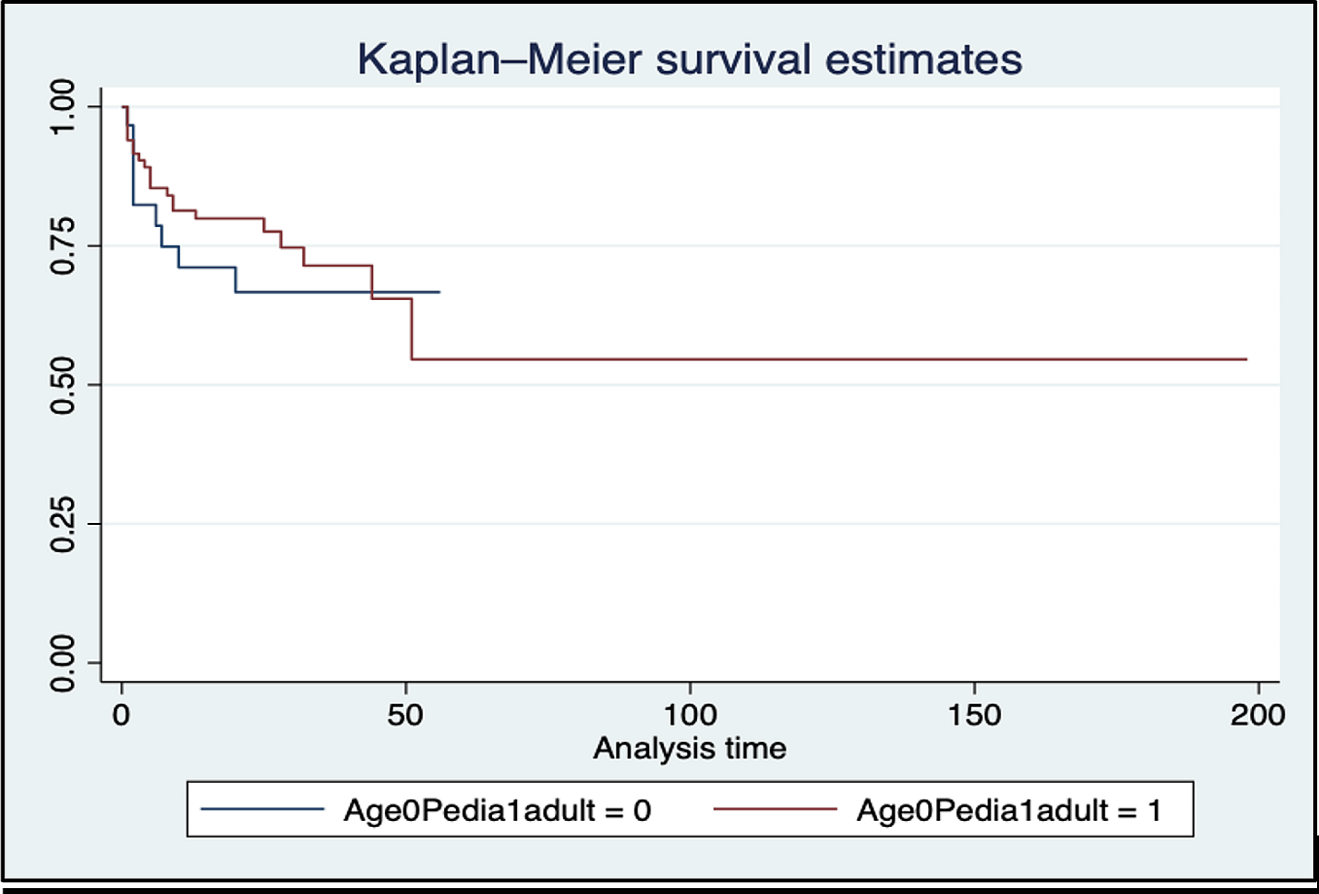
Kaplan-Meier curve on survival of adult and pediatric tetanus patients seen in a tertiary hospital in the Philippines

Septic shock was the most common cause of death in adults (33%, *n* = 8), while respiratory failure was the most common cause of death in children (40%, *n* = 4). Survival analysis using the Kaplan-Meier curve showed better survival among adults until day 45 to 50 (Fig. 2). However, a log-rank test showed no significant difference between adult and pediatric subgroups in terms of survival.

**Table 1:** Clinical characteristics and outcomes of non-neonatal tetanus patients seen in a tertiary hospital in the Philippines

Parameter	**Group** *n* (%)*		
	**All patients** (*n* = 138)	Adult (*n* = 98)	Pediatric (*n* = 40)
**Demographics**			
Age (median, range)	37.5 (2–75)	47 (20–75)	8 (2–18)
Sex			
Male	102 (74%)	72 (73%)	30 (75%)
Female	36 (26%)	26 (27%)	10 (25%)
Place of Residence			
National Capital Region (NCR)	36 (41%)	30 (46%)	6 (26%)
Region III	8 (9%)	6 (9%)	2 (9%)
CALABARZON	41 (47%)	26 (40%)	15 (65%)
Others	3 (3%)	3 (5%)	0 (0%)
No available data	50		
**Risk Factors**			
Comorbidities			
Malignancy	16 (18%)	16 (23%)	0 (0%)
Hypertension or other cardiovascular disease	12 (13%)	12 (17%)	0 (0%)
Pulmonary disease	11 (12%)	10 (14%)	1 (5%)
Diabetes mellitus or other endocrine disease	8 (9%)	8 (11%)	0 (0%)
Stroke or other neurologic disease	6 (7%)	6 (9%)	0 (0%)
Renal disease	2 (2%)	2 (3%)	0 (0%)
Hepatic disease	1 (1%)	1 (1%)	0 (0%)
No available data	48		
Vaccination Status	-	-	-
**Clinical Presentation**			
Mode of Infection			
Post-traumatic	45 (49%)	32 (46%)	13 (59%)
Post-infectious	35 (38%)	29 (42%)	6 (27%)
Post-surgical	3 (3%)	3 (4%)	0 (0%)
Unknown	8 (9%)	5 (7%)	3 (14%)
No available data	47		
Type of Injury Sustained			
Puncture wound	29 (32%)	24 (35%)	5 (23%)
Abrasion	7 (8%)	4 (6%)	3 (14%)
Head and neck infection	8 (9%)	1 (1%)	7 (32%)
Operative wound	3 (3%)	3 (4%)	0 (0%)
Dental manipulation	2 (2%)	2 (3%)	0 (0%)
Animal bite	1 (1%)	1 (1%)	0 (0%)
Burn	1 (1%)	1 (1%)	0 (0%)
Foreign body aspiration or insertion	1 (1%)	1 (1%)	0 (0%)
Others	39 (43%)	32 (46%)	7 (32%)
No available data	47		
Site of Injury or Infection			
Lower extremities	38 (42%)	33 (48%)	5 (23%)
Head	21 (23%)	11 (16%)	10 (45%)
Trunk	15 (16%)	13 (19%)	2 (9%)
Upper extremities	4 (4%)	2 (3%)	2 (9%)
Perineal area	3 (3%)	3 (4%)	0 (0%)
Others	10 (11%)	7 (10%)	3 (14%)
No available data	47		
Interval from Trauma to First Symptom, in days (median, range)	7 (0-176)	8 (0-176)	3.5 (1–12)
Interval from First Symptom to Spasm, in days (median, range)	1 (0–28)	1 (0–28)	1 (0–3)
Initial or Presenting Symptom			
Trismus	55 (65%)	43 (67%)	12 (60%)
Dysphagia	9 (11%)	8 (13%)	1 (5%)
Spasms	9 (11%)	6 (9%)	3 (15%)
Rigidity	3 (4%)	1 (2%)	2 (10%)
Neck stiffness	8 (10%)	6 (9%)	2 (10%)
No available data	54		
Symptoms			
Trismus	83 (99%)	63 (98%)	20 (100%)
Spasms	72 (86%)	53 (83%)	19 (95%)
Dysphagia	49 (58%)	41 (64%)	8 (40%)
Rigidity	47 (56%)	36 (56%)	11 (55%)
Neck stiffness	34 (40%)	27 (42%)	7 (35%)
Risus sardonicus	17 (20%)	12 (19%)	5 (25%)
Opisthotonos	16 (19%)	9 (14%)	7 (35%)
Spasticity	11 (13%)	5 (8%)	6 (30%)
No available data	54		
Number of Body Segments with Spasms (median, range)	2 (0–4)	1 (0–4)	2 (0–4)
Body Segments Affected with Spasms			
Head	19 (23%)	15 (23%)	4 (20%)
Trunk, including abdomen	58 (69%)	44 (69%)	14 (70%)
Arms	27 (32%)	17 (27%)	10 (50%)
Legs	39 (46%)	27 (42%)	12 (60%)
No available data	54		
Type of Tetanus			
Generalized	113 (86%)	81 (85%)	32 (86%) 1 (3%)
Localized	7 (5%)	6 (6%)	
No available data	6		
Stage			
I	6 (5%)	5 (5%)	1 (3%)
II	39 (30%)	32 (34%)	7 (19%)
III	76 (58%)	49 (52%)	27 (73%)
No available data	6		
**Management**			
Tetanus Antitoxin (ATS) Dose, in units (median, range)	10,000 (1,000–40,000)	10,000 (3,000–40,000)	3,000 (1,000–10,000)
Route of Administration of ATS			
IM alone	66 (80%)	49 (79%)	17 (85%)
IM + wound infiltration	11 (13%)	9 (15%)	2 (10%)
Not specified	5 (6%)	4 (6%)	1 (5%)
No available data	56		
TeAna Dose, in ml	0.5	0.5	0.5
Medications			
Metronidazole	91 (100%)	71 (100%)	20 (100%)
Diazepam	85 (93%)	69 (97%)	16 (80%)
Midazolam	44 (48%)	25 (35%)	19 (95%)
Rocuronium	25 (27%)	8 (11%)	17 (85%)
Magnesium sulfate	42 (46%)	36 (51%)	6 (30%)
Baclofen	53 (58%)	53 (75%)	0 (0%)
Carbamazepine	20 (22%)	20 (28%)	0 (0%)
No available data	47		
Primary Service			
Adult Neurology	89 (64%)	89 (91%)	0 (0%)
Surgery	4 (3%)	3 (3%)	1 (2%)
Pediatrics	39 (28%)	0 (0%)	39 (98%)
Services Onboard			
ORL	76 (90%)	56 (88%)	20 (100%)
General Medicine	45 (54%)	45 (70%)	0 (0%)
IDS	74 (88%)	58 (91%)	16 (80%)
Pulmonology	32 (38%)	32 (50%)	0 (0%)
Cardiology	20 (24%)	8 (13%)	12 (60%)
Nephrology	22 (26%)	20 (31%)	2 (10%)
Medical Oncology	7 (8%)	7 (11%)	0 (0%)
Surgery	34 (40%)	30 (47%)	4 (20%)
Rehabilitation Medicine	40 (48%)	29 (45%)	11 (55%)
Number of Services Onboard (median, range)	6 (2–13)	6 (2–13)	4 (3–8)
**Clinical Course**			
Duration of Hospital Stay, in days (median, range)	19 (0-198)	19 (0-198)	20 (0–56)
Duration of ICU Stay, in days (median, range)	10 (0–49)	11.5 (0–49)	6 (0–47)
Interval from Admission until Tracheostomy, in days (median, range)	0 (0-169)	0 (0-169)	0 (0–1)
Number of Complications During Admission (median, range)	1 (0–7)	1 (0–7)	2 (0–5)
Complications			
Pneumonia	54 (59%)	43 (62%)	11 (50%)
Catheter-associated urinary tract infection	10 (11%)	5 (7%)	5 (23%)
Bacteremia	12 (13%)	10 (14%)	2 (9%)
Catheter-related bloodstream infection	3 (3%)	2 (3%)	1 (5%)
Acute kidney injury from rhabdomyolysis	6 (7%)	5 (7%)	1 (5%)
Acute kidney injury from other causes	11 (12%)	10 (14%)	1 (5%)
Pulmonary embolism or other embolic events	3 (3%)	3 (4%)	0 (0%)
Bleeding from any cause	13 (14%)	8 (12%)	5 (23%)
Pressure ulcers	12 (13%)	6 (9%)	6 (27%)
No available data	47		
Presence of Autonomic Dysfunction			
Yes	21 (23%)	14 (20%)	7 (32%)
No	71 (77%)	56 (80%)	15 (68%)
No available data	46		
**Outcome**			
Outcome of Admission			
Discharged	78 (67%)	61 (69%)	17 (61%)
Expired	34 (29%)	24 (27%)	10 (36%)
Home against medical advice	5 (4%)	4 (4%)	1 (4%)
No available data	21		
Cause of Mortality, If Applicable			
Septic shock	9 (26%)	8 (33%)	1 (10%)
Cardiogenic shock	3 (9%)	3 (13%)	0 (0%)
Obstructive shock	1 (3%)	1 (4%)	0 (0%)
Respiratory failure	6 (18%)	2 (8%)	4 (40%)
Fatal arrhythmia	6 (18%)	4 (17%)	2 (20%)
Others	3 (9%)	2 (8%)	1 (10%)
Unspecified	6 (18%)	4 (17%)	2 (20%)

*Proportions were computed based on the available data per category

### Factors associated with survival of tetanus patients

Each variable noted with p-value less than 0.2 between the pediatric and adult subgroups were checked as possible factors for survival. All were analyzed individually using simple cox regression analysis. Accordingly, hypertension increased the hazard of death by 4.5 times (*p* = 0.004) while administration of MgSO4 reduced hazard by 9 times. However, controlling for possible confounders and effect modifiers using multiple cox regression analysis, only MgSO4 significantly decreased the hazard by 35 times compared to patients who had no MgSO4 (*p* = 0.005) (see Table 2).

**Table 2:** Factors possibly associated with survival of tetanus patients and their hazard ratio (HR) using log rank test and cox regression analysis

Parameter	HR (simple cox regression)	*p*-value	HR (cox Regression)	*p*-value
Age	0.78	0.54	2.9	0.2
Hypertension	4.5	0.004	3.97	0.17
Malignancy	0.83	0.77		
Type of injury (puncture wound)	1.37	0.68		
Site (legs)	6.5	0.13	1.3	0.26
Incubation period	1.0	0.77		
Days to spasm	0.62	0.07	0.5	0.08
Stage (3)	2.08	0.48		
Midazolam	0.65	0.34		
Rocuronium	0.76	0.62		
Magnesium sulfate	0.11	0.005	0.02	0.005
Baclofen	0.6	0.24		
Primary service (Adult Neurology)	0.9	0.93		
Number of services	0.84	0.16	0.67	0.096
CAUTI	0.63	0.54		
Pressure ulcer	0.21	0.13	1.7	0.35

## Discussion

As of 2022, the global incidence of tetanus was at 0.9 per 1 million total population, representing a steady decline since 2012 when the incidence was at 2 per 1 million total population. The Western Pacific region ranks second with an incidence of 0.6 per 1 million total population, of which the Philippines has the highest incidence rate of approximately 7 per 1 million total population [4, 6]. This study found an incidence rate of 0.03% or 269 per 1 million inpatient admissions in a tertiary hospital. The significantly higher incidence noted in this study is likely due to referral bias since UP-PGH serves as the national referral center for tertiary care in the country. Similar to Philippine data, this study showed a gradual decrease in tetanus cases over the past decade [8].

A systematic analysis on the global epidemiology and burden of tetanus over the past 30 years found that the incidence of tetanus was significantly correlated with economic level, and has also been termed as a “disease of the underdeveloped world [3, 9].” Agricultural lifestyles, humid environments, poor wound care, low vaccination rates, and poor awareness of tetanus are some factors which could explain the regional burden of tetanus [10]. Despite the inclusion of free tetanus vaccination for children under the Expanded Program on Immunization [11], there are still low tetanus immunization rates locally, with fluctuating immunization coverage ranging from 55 to 81% over the past ten years [12]. Data on catch-up immunization rates in both the pediatric and adult population are also lacking. This is likely due to the devolved healthcare service delivery and out-of-pocket expenditure for adult tetanus immunization.

Similar to the findings of two earlier retrospective studies done on adult tetanus patients from 1997 to 2003 and from 2008 to 2013 in the same institution, the majority of tetanus patients are males regardless of age group, with unknown immunization status [13, 14]. The presence of comorbidities which was not explored in these previous studies revealed that more 1 in 5 adult tetanus patients had concomitant malignancy, and that interestingly, chronic hypertension significantly increased the hazard of death by 4.5 times compared to those who are non-hypertensives. This may be due to the interaction between an already compromised cardiovascular status and autonomic dysfunction. Dysautonomia, a feature that is usually associated with severe tetanus, has been noted in 32-62.1% of patients with tetanus [15, 16]. In a 10-year retrospective cross-sectional study done in two tertiary level centers in Pakistan involving 96 tetanus patients, those presenting with autonomic disturbance were found to have a higher mortality rate (47%) compared to those without autonomic dysfunction (15%; *p* = 0.002) [16]. The link between the propensity towards poor outcomes and the interaction between hypertension and autonomic dysfunction has been emerging in the literature. In a systematic review involving 17 studies, it has been proposed that patients with chronic hypertension have increased arterial stiffness and consequently a reduced baroreflex sensitivity [17]. This implies that any form of autonomic dysfunction, as in the case of tetanus, could be more deleterious among hypertensives compared to those without chronic hypertension. It is interesting to note however that no significant relationship has been found between dysautonomia and survival in the results of this study; this may be attributed to poor detection and documentation.

While tetanus is known to arise from post-traumatic wound contamination [2, 3], this study noted differences in terms of the likely source of infection between adults and children. Tetanus was mostly acquired through puncture wounds on the lower extremities for adults, while head and neck infections were the more common presumed source in children. Two retrospective studies on pediatric tetanus found trauma to still be the most common cause of tetanus among children, but noted otogenic and dental infections to be the next likely etiology (52% vs. 44% and 42% vs. 38% in these studies respectively) [18, 19]. Such differences may be explained by their usual activities, as adults are more exposed to external injuries from occupational hazards, whereas children are more prone to develop otitis media and dental infection due to differences in ear anatomy, poorer hygiene, and the lack of a fully mature immune system [20].

Trismus was still the most common presenting symptom, followed by dysphagia and truncal spasms, consistent with existing literature [2, 13, 14]. On chart review, it was observed that patients often opted to observe their symptoms for a few days prior to seeking consult, while some consulted with a dentist, an otorhinolaryngologist, or the local health center who failed to recognize tetanus and sent these patients home. Educating the public as well as encouraging these professionals to have a higher index of suspicion for tetanus can potentially minimize delays in management, and consequently decrease the risk of further morbidity and mortality in these patients.

Metronidazole was the antibiotic of choice for all patients, consistent with existing guidelines [1]. Diazepam was given in most patients for control of spasms, but midazolam and rocuronium were used more in the pediatric subgroup. A recent review on the pharmacological management of tetanus noted that although midazolam is theoretically better than diazepam due to its potency and faster onset of action, substantial evidence showing the efficacy of midazolam or its superiority over diazepam in tetanus is still limited [21]. The use of rocuronium in tetanus patients has been documented in a few case reports in adults, but data on its efficacy in the pediatric population is still lacking [20].

In terms of factors associated with survival, this study found that treatment with magnesium sulfate significantly decreased the hazard of death by 35 times (*p* = 0.005). The role of magnesium sulfate in the treatment of tetanus is based on its mechanism of action as a presynaptic neuromuscular blocker via calcium ion antagonism thereby reducing muscle spasms, and as an inhibitor of catecholamine release thereby reducing the tendency for hypertensive spikes and dysautonomia [23]. It is logical to consider that its role in reducing mortality could be due to its role in reducing autonomic dysfunction in patients with tetanus. This relationship is also reflected in a prospective study done in a tertiary institution in Kolkata, India involving 86 patients which showed a lower rate of dysautonomia among those treated with intravenous magnesium sulfate (10%) compared to those who did not receive the drug (24%) [24]. Although there is significant benefit in terms of spasm control, no mortality benefit has been demonstrated. A randomized controlled trial involving 42 patients demonstrated that patients who received magnesium encountered significant reductions in spasm frequency (4.1 ± 1.6 spasms per hour at the onset to 0.13 ± 0.05 spasms per hour in the second week of trial) compared to those who received diazepam-only treatment (from 3.4 ± 1.5 spasms per hour to 0.33 ± 0.2 spasms per hour, *p* = 0.010) [25]. These findings are also congruent with the results of a randomized trial in a tertiary center in Pakistan involving 36 patients [26]. The non-demonstration of significant survival benefit of magnesium sulfate in the cited trials could be due to a dose-response issue or a type 2 error due to low sample size. In a retrospective study done in Nepal comparing small and large doses of magnesium sulfate among patients with tetanus, those who received larger doses had lower mortality rates compared to the lower dose group [27].> Further studies involving larger sample sizes and various dosing regimens are needed to demonstrate the potential efficacy of magnesium sulfate in reducing mortality among patients with tetanus.

The Philippines reported a case fatality rate of 22% for non-neonatal tetanus as of 2021. Earlier reviews done in UP-PGH reported a case fatality rate of 22.2% from 1997 to 2023, and 19% from 2008 to 2013 [13, 14]. This study noted a higher overall case-fatality rate of 29%, 24% in adults and 36% in the pediatric subgroup. Age greater than 60 years, incubation period, higher severity staging, and presence of tachycardia on admission have been previously identified to be associated with increased mortality in tetanus [13, 14, 28]. Although not found to be statistically significant, the higher proportion of patients with stage III tetanus (58% vs. 56% and 28%) and the shorter incubation periods (7 days vs. 16 days) may explain the higher mortality rates noted in this study [13, 14]. The shorter incubation period among children (median: 3.5 days) compared to adults (median: 8 days) may also be associated with their higher mortality rate. Septic shock was the leading cause of death among tetanus patients, mostly secondary to nosocomial pneumonia and bacteremia which were the leading complications encountered during hospitalization.

This study has several limitations. Given its retrospective nature, data collection was primarily limited by the availability and completeness of existing records; hence, pertinent information such as vaccination status which were not documented in patient charts could no longer be retrieved. However, despite this limitation, the total number of available charts and the number of years covered (i.e. minimum of 82 samples per category over 12.5 years) are still greater than those of previous retrospective studies from the same institution, as well as other cited studies from different institutions and countries, with sample sizes ranging from 24 to 54 over 1 to 6 years [13, 14, 18, 19]. Possible reporting bias was avoided by disclosing the number of unavailable data per category, and computing proportions based on what was available. The risk of measurement bias was also minimized by having two reviewers extract data independently into a pre-made detailed abstraction form. Another limitation is that this study was confined to a single center with cases mainly coming from its nearby regions. Nevertheless, being the largest tertiary referral hospital in the country with a large sample size, these results are still likely reflective of tetanus cases in the Philippines.

## Conclusions

Tetanus remains to have a high incidence and case fatality rate in the Philippines. Although the total number of cases and deaths from tetanus have been steadily decreasing over the past decade, its high morbidity and mortality rate demands greater efforts to address this vaccine-preventable disease. As immunization remains to be the cornerstone of tetanus prevention, the need to address the low immunization rates especially in adults and at-risk individuals becomes apparent. Increasing vaccination coverage, improving public awareness, and educating other health professionals who serve as the initial point of contact of these patients with the healthcare system can potentially minimize delays in initiating the appropriate management, and consequently decrease mortality. Results of this study also support the use of magnesium sulfate to improve overall survival among tetanus patients.

To our knowledge, this is the largest retrospective cross-sectional study on the clinical profile and outcomes of both adult and pediatric non-neonatal tetanus patients in the Philippines. Future research on tetanus can prospectively explore the association between chronic hypertension, the use of magnesium sulfate, and outcomes of tetanus patients. Future studies may also concentrate on the impact of immunization efforts on tetanus prevalence. Findings from this study provide insights on the risk factors, clinical presentation, and predictors of mortality in tetanus patients, which can potentially contribute to overall improvement in the management of tetanus. Ultimately, this study can serve as a springboard for the development of future programs and policies focused on reducing morbidity and mortality from tetanus in the Philippines and in other developing countries.

## Data Availability

The datasets used and analyzed during the current study are available from the corresponding author on reasonable request.
